# Identification of a novel association for the WWOX/HIF1A axis with gestational diabetes mellitus (GDM)

**DOI:** 10.7717/peerj.10604

**Published:** 2021-01-14

**Authors:** Izabela Baryla, Elzbieta Pluciennik, Katarzyna Kośla, Marzena Wojcik, Andrzej Zieleniak, Monika Zurawska-Klis, Katarzyna Cypryk, Lucyna Alicja Wozniak, Andrzej K Bednarek

**Affiliations:** 1 Department of Molecular Carcinogenesis, Medical University of Lodz, Lodz, Poland; 2 Department of Structural Biology, Medical University of Lodz, Lodz, Poland; 3 Department of Internal Diseases and Diabetology, Medical University of Lodz, Lodz, Poland

**Keywords:** Gestational diabetes mellitus (GDM), Glycolysis, Hypoxia-inducible factor 1α (HIF1α), WW domain-containing oxidoreductase (WWOX)

## Abstract

**Background:**

Although the WW-domain-containing oxidoreductase (WWOX)/Hypoxia-inducible factor 1 (HIF1) pathway is a well-known regulator of cellular glucose and energy metabolism in pathophysiological processes, its role in gestational diabetes mellitus (GDM), remains elusive. We undertook this study to determine the effect of WWOX/HIF1A signaling on the expression of glucose metabolism genes in GDM patients.

**Methods:**

Leukocytes were obtained from 135 pregnant women with (*n* = 98) or without (*n* = 37) GDM and, in turn, 3 months (*n* = 8) and 1 year (*n* = 12) postpartum. Quantitative RT-PCR was performed to determine gene expression profiles of the WWOX/HIF1A-related genes, including those involved in glucose transport (*SLC2A1, SLC2A4*), glycolytic pathway (*HK2, PKM2, PFK, LDHA*), Wnt pathway (*DVL2, CTNNB1*), and inflammatory response (*NFKB1*).

**Results:**

GDM patients displayed a significant downregulation of *WWOX* with simultaneous upregulation of *HIF1A* which resulted in approximately six times reduction in *WWOX/HIF1A* ratio. As a consequence, *HIF1A* induced genes (*SLC2A1, HK2, PFK, PKM*) were found to be overexpressed in GDM compared to normal pregnancy and negative correlate with *WWOX/HIF1A* ratio. The postpartum *WWOX* expression was higher than during GDM, but its level was comparable to that observed in normal pregnancy.

**Conclusions:**

The obtained results suggest a significant contribution of the *WWOX* gene to glucose metabolism in patients with gestational diabetes. Decreased *WWOX* expression in GDM compared to normal pregnancy, and in particular reduction of *WWOX/HIF1A* ratio, indicate that WWOX modulates HIF1α activity in normal tissues as described in the tumor. The effect of HIF1α excessive activation is to increase the expression of genes encoding proteins directly involved in the glycolysis which may lead to pathological changes in glucose metabolism observed in gestational diabetes.

## Introduction

Gestational Diabetes Mellitus (GDM) is a carbohydrate intolerance with onset or first recognition during pregnancy that complicates 1–22% of all pregnancies, depending on the ethnic background of the study population and the diagnostic criteria used (Coustan et al., 2010; Jenum et al., 2012). As per worldwide trend, the incidence of GDM in Poland is rising, from 4.67% in 2010 to 7.45% in 2012, becoming an important health issue (Wierzba et al., 2017). Importantly, the presence of GDM not only increases the risk of serious maternal and perinatal complications, but also the subsequent development of diabetes mellitus type 2 (T2DM), metabolic syndrome, and cardiovascular disease in the mother and her child (Metzger, 2007; Shah, Retnakaran & Booth, 2008; Bellamy et al., 2009). It has recently been also reported that a history of GDM is associated with the development of endometrial cancer (Wartko et al., 2017). GDM has a complex pathology that is characterized by glucose intolerance and insulin resistance. Despite this, there is no consensus in the underlying pathophysiology events during diabetic pregnancy. In this context, the pro-inflammatory state and exaggerated oxidative stress have been proposed as important pathological contributors (Lappas et al., 2011; Gomes et al., 2013).

Hypoxia is a derangement of oxygen homeostasis which plays an important role in metabolism and survival. Hypoxia-inducible factor 1α (HIF-1α), encoded by the *HIF1A* gene, is a key mediator of cellular adaptive responses to hypoxia that regulates a transcriptional activity of numerous target genes, including those involved in glucose transport and metabolism, among others. It is now well-recognized that HIF1α upregulation has a beneficial effect on a normal placental differentiation and embryonic development in early pregnancy, thus, when the feto-placental unit is developing under hypoxic conditions (Caniggia et al., 2000; Wakeland et al., 2017), whereas activation of this transcription factor during late pregnancy may contribute to adverse pregnancy outcomes (Li, Chen & Li, 2013; Albers et al., 2019). HIF1α expression associated with VEGF activation is related both with placenta formation and obesity (Messineo et al., 2016) that may contribute to prolonged HIF1A high expression in GDM patients. In the context diabetes, the role of hyperglycemia in the regulation of the HIF1α signaling is controversial and remains the subject to much debate. On the one hand, high glucose concentration has been shown to impair hypoxia-induced stabilization and the transcriptional activation function of HIF-1α in cultured human dermal fibroblasts and human dermal microvascular endothelial cells, suggesting that hyperglycemia results in the loss of cellular adaptation to low oxygen in diabetes (Catrina et al., 2004). Additionally, lower HIF-1α protein levels were found in biopsies obtained from foot ulcers of diabetic patients (Catrina et al., 2004). On the other hand, high glucose-induced upregulation of HIF-1 *α* and its target genes in human mesangial cells has been proposed as an important mechanism underlying the development of diabetic glomerulopathy (Isoe et al., 2010). Despite these advances, knowledge of the importance of the HIF-1α pathway in diabetic pregnancy is still limited. In this regard, the association of GDM with an excessive chronic hypoxia stress accompanied by the inflammatory response has been documented in murine placentas (Li, Chen & Li, 2013).

The WW domain-containing oxidoreductase (*WWOX)* gene is located on 16q23.3–24.1, it spans the *FRA16D*, one of the most common fragile sites (Ludes-Meyers et al., 2003). *WWOX* has been identified as a tumor suppressor (Bednarek et al., 2001) with a reduced expression in numerous human tumors (Bednarek et al., 2000; Nunez et al., 2005; Płuciennik et al., 2006; Qin et al., 2006; Paige et al., 2010). Mounting evidence points to an important role of WWOX in metabolic homeostasis. Indeed, it has been shown that *Wwox*-knockout (KO) mice display hypoglycemic, hypotriglyceridemic, and hypercholesterolemic phenotypes (Aqeilan et al., 2008). Furthermore, in cellular and animal models, WWOX has been proved to be able to regulate glucose metabolism through suppression of aerobic glycolysis, as a result of its inhibitory action on HIF1α (Abu-Remaileh & Aqeilan, 2014). More recently, skeletal muscle-specific deletion of *Wwox* has been reported to impair mitochondrial glucose oxidation and stimulate lactate production, leading to disruption of whole-body glucose homeostasis. Importantly, *Wwox* deficiency was accompanied by enhanced expression of HIF1α and its target genes, including those encoding key glycolytic enzymes (Abu-Remaileh et al., 2019).

To date, there is a paucity of data available examining the effect of GDM on the WWOX/HIF1 signaling pathway in human leukocytes. Therefore, the main purpose of this study was to investigate whether there is transcriptional dysregulation of the selected WWOX/HIF1-related genes in leukocyte obtained from clinically well-characterized diabetic patients at the time of GDM diagnosis and the postpartum period (3 months and one year after delivery). Out of numerous genes related to the WWOX/HIF1 axis, we chose those involved in glucose transport [glucose transporters 1 (*SLC2A1*) and 4 (*SLC2A4*)]; glycolytic pathway [hexokinase 2 (*HK2*), phosphofructokinase (*PFK*), pyruvate kinase muscle isozyme M2 (*PKM2*), and lactate dehydrogenase A(*LDHA*)]; the Wnt signaling pathway [dishevelled segment polarity protein 2 (*DVL2*) and catenin beta 1 (*CTNNB1*)]; and inflammatory response [nuclear factor kappa B subunit 1 (*NFKB1*)]. We also looked for potential gene-gene expression correlations as well as associations between the expression of the aforementioned genes and clinical phenotypes of the patients. In this study, we used leukocytes as the experimental model since these cells are a well-known drivers of the inflammatory process that is closely linked to diabetic pregnancy as well as the WWOX/HIF signaling pathway. In addition, the ability of leukocytes to reflect pathological changes elsewhere in the body have been suggested in the literature (Liew et al., 2006). In support of this concept, leukocyte gene expression profiling has been successfully applied to GDM to understand the molecular aspects of this disease (Mac-Marcjanek et al., 2018). It is noteworthy that the peripheral blood is a convenient source of leukocytes for this study, allowing circumvent ethical concerns linked to the invasive procedures needed to take other types of tissue samples from pregnant women.

## Materials & Methods

### Study participants

A total of 135 Caucasian pregnant women consisted of 37 women with normal glucose tolerance (NGT) and 98 women with GDM were enrolled and studied at the Outpatient Diabetological Clinic in Lodz, Poland. There were three visits of the patients to the clinic: the first during the third trimester of pregnancy (at 24–28 weeks’ gestation or later if it was not possible during this period) when the patients were diagnosed for GDM, followed by the postpartum second and third visits at 3 months and one year, respectively. During the first visit, a one-step 75 g oral glucose tolerance test (OGTT) was performed among all pregnant women. GDM was diagnosed according to the Polish Diabetes Association (PDA)/WHO criteria when at least one value of the venous plasma glucose concentrations was equal or exceeded the thresholds of 92 mg/dL (5.1 mmol/L) for fasting, 180 mg/dL (10.0 mmol/L) for 1 h post-glucose load, or 153 mg/dL (8.5 mmol/L) for 2 h post-glucose load (Zalecenia kliniczne dotyczące postępowania u chorych na cukrzycę, 2014). The pregnant NGT women had a negative screen. The inclusion criteria were: Caucasian ethnic background and age >18 years. None of the participants had family history of diabetes in first-degree relatives, GDM in a previous pregnancy, diabetes diagnosed prior pregnancy, systemic infectious, and took any drugs known to affect carbohydrate metabolism.

**Figure 1:**
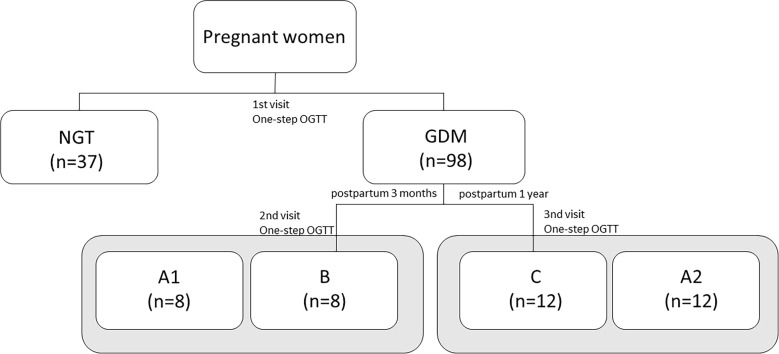
The classification of subjects to the study groups during diabetic pregnancy and the postpartum period based on the OGTT results.

After delivery, only a small group of women with prior GDM returned for the second (3 month) and third (1 year) study visits, where they were screened for prediabetes with the OGTT, according to the PDA criteria. Prediabetes was recognized either through fasting plasma glucose (FPG) between 100 and 125 mg/dl (i.e., impaired fasting glucose, IFG) or through the blood glucose level between 140 and 199 mg/dl (i.e., impaired glucose tolerance, IGT) at 2-h OGTT (Zalecenia kliniczne dotyczące postępowania u chorych na cukrzycę, 2014). The women at 3 months and 1 year postpartum were assigned to the group B and group C, respectively. The procedure for assigning patients to the appropriate study groups is shown in the Fig. 1.

Of note, because GDM women had not been in receipt of any therapy at the time of inclusion into the study, changes in their metabolic parameters are a result of solely the disease.

All clinical investigations were conducted in accordance with the guidelines of The Declaration of Helsinki, and approved by the Ethical Committee of the Medical University of Lodz (No. RNN/186/11/KE from 20.09.2011 with changes KE/596/15 from 21.04.2015 and RNN/676/14/KB from 23.09.2014). Informed written consent was obtained from all participants.

### Clinical and biochemical data collection

The patients underwent clinical and laboratory assessment at GDM diagnosis (*n* = 135) and 3 months (group B; *n* = 8) and 1 year (group C; *n* = 12) postpartum. Maternal age and pre-pregnancy weight were self-reported by all participants. Pregnancy height and weight were measured at the first visit using standardized procedures and calibrated equipment. Body mass index (BMI) was calculated by dividing the weight in kilograms by the height in meters squared. Gestational age was established based on the last menstrual period. Systolic (SBP) and diastolic (DBP) blood pressures were measured after 10 min of rest in a sitting position using an electronic monitor. For biochemical analysis, venous blood samples were collected from the patients during pregnancy and postpartum, in all cases following a 12 h overnight fast. Plasma glucose concentration was analyzed by the glucose oxidase method. Plasma triglycerides (TGs), and HDL- and LDL-cholesterol (HDL-C and LDL-C) concentrations were determined by enzymatic colorimetric methods with triglyceride GPO-PAP and the total cholesterol CHOD-PAP kits (Roche Diagnostics GmbH, Germany). The glycosylated hemoglobin A1C (HbA1C) was measured by a latex enhanced turbidimetric immunoassay using specific monoclonal antibodies. The concentration of C-reactive protein (CRP) was determined by turbidimetric assay using the cassette COBAS INTEGRA C-Reactive Protein (Latex, Roche Diagnostics GmbH, Germany). The biochemical assays were carried out on COBAS INTEGRA analyzer (Roche, SA). Plasma insulin was quantified using Elecsys insulin assay (Roche Diagnostics GmbH, Germany).

### Insulin resistance/sensitivity and insulin secretion indices

The homeostasis model assessment of insulin resistance (HOMA-IR; Eq. (1)) and β-cell function (HOMA-B; Eq. (2)) were calculated, respectively (Matthews et al., 1985):

(1) \begin{eqnarray*}& & \mathrm{HOMA - IR}=[\text{fasting insulin}~(\mathrm{\mu }\mathrm{U/ mL})\times \text{fasting glucose (mg/dL)}]/405\end{eqnarray*}

(2) \begin{eqnarray*}& & \mathrm{HOMA - B}=[360\times \text{fasting insulin}~(\mathrm{\mu }\mathrm{U/ mL})]/[\text{fasting glucose}\mathrm{(mg/ dL)}-63].\end{eqnarray*}

The quantitative insulin sensitivity check index (QUICKI-IS) was calculated from the Eq. (3) (Katz et al., 2000):

(3) \begin{eqnarray*}\mathrm{QUICKI - IS}=1/\{\mathrm{log}[\text{fasting insulin}~(\mathrm{\mu }\text{U/mL})]+\mathrm{log}[\text{fasting glucose (mg/dL)}]\}.\end{eqnarray*}

### Leukocytes separation and RNA extraction

Leukocytes were separated from fresh anticoagulated blood samples (10 mL) of each patient as described elsewhere (Wojcik et al., 2016; Wojcik et al., 2014). In brief, blood samples were centrifuged at 3,000 rpm for 10 min at 4 °C, and then the plasma supernatants were discarded. After adding red blood cell lysis buffer (0.5 M NH_4_Cl, 10 mM KHCO_3_, 10 mM EDTA, pH 8.0) and 30 min incubation on ice, the samples were centrifuged at 4,000 rpm for 10 min at 4 °C. The pellets containing leukocytes were washed with the phosphate-buffered saline (PBS). Total RNA was isolated from leukocytes using a commercially available TRI Reagent (Sigma-Aldrich, USA). After filtering on clean-up columns (Syngen, Poland), RNA quantity and quality were assessed with a BioDrop UV/VIS Spectrophotometer (SERVA, Germany) at UV_260_ and UV_260∕280_, respectively.

### Quantitative RT-PCR

Quantitative RT-PCR (RT-qPCR) was performed for all the studied genes (*WWOX, HIF1A*, *NFKB1, SLC2A1, SLC2A4*, *HK2, PKM2, PFK, LDHA*, *DVL2,* and *CTNNB1*) at the time of GDM diagnosis (the first visit) and 3 months and one years after delivery (the second and third visits, respectively). For this purpose, cDNA was produced from 5 µg of each high-quality total RNA sample using the ImProm-II™ Reverse Transcription System (Promega, USA). The resulting cDNA was subjected to RT-qPCR using forward and reverse primers and GoTaq qPCR master mix (Promega, USA). For each gene analyzed, a specific primers pair was designed using PrimerQuest^®^Tool. The sequences of primers, size of the product, and GeneBank accession number are given in Table 1. Reactions were performed in duplicate on a LightCycler 480 II (Roche Diagnostics GmbH, Germany) with initial denaturation at 95  °C for 2 min, followed by 40 cycles of 95  °C for 30 s and proper annealing temperature for 30 s. The amplification of specific transcript was confirmed by melting curve at the end of each PCR. The Ribosomal Protein Lateral Stalk Subunit P0 (*RPLPO),* 40S ribosomal protein S17 (*RPS17*), and H3 Histone Family Member 3A (*H3F3A*) were used as the housekeeping genes for internal normalization. The Universal Human Reference RNA (Agilent, USA) was used as a calibrator. The relative expression of the target genes was calculated using the CT value according to the Pfaffl method (Pfaffl, 2001) with the geometric mean of Ct values for three reference genes was used as a normalization factor.

### Statistical analysis

Variables are presented as median values with 25–75% interquartile range. The distribution of analyzed biochemical and expression data was checked by the Shapiro–Wilk test. Differences between variables were calculated using the Mann–Whitney *U* test. The Wilcoxon matched-pairs signed rank test was used to assess differences in the matched pairs data of the patients at different time intervals during the research. The Spearman rank correlation was applied to investigate correlations. A *p*-value <0.05 was considered as significant. Statistical analyses were performed using GraphPad Prism 5.1 software and R Studio.

**Table 1:** RT-PCR primer sequences.

**Gene**	**Description**	**ID gene**	**Sequence (5′**→**3′)**	**Product length (bp)**	**Annealing temperature** (°C)
*WWOX*	WW domain containing oxidoreductase	NM_016373.3	I step F: TGCAACATCCTCTTCTCCAACGAGCTGCAC R: TCCCTGTTGCATGGACTTGGTGAAAGGC II step F: GAGCTGCACCGTCGCCTCTCCCCAC R: TCCCTGTTGCATGGACTTGGTGAAAGGC		
				171	63
	
				150	63
	
*HIF1A*	Hypoxia inducible factor 1 subunit alpha	NM_001530.3	F: CCGGCGGCGCGAACGACAAG R: TGCGAACTCACATTATGTGG	148	58
*SLC2A1*	Solute carrier family 2 member 1	NM_006516.3	F: CTTCACTGTCGTGTCGCTGT R: TGAGTATGGCACAACCCGC	95	58
*SLC2A4*	Solute carrier family 2 member 4	NM_001042.2	F: CCTGCCAGAAAGAGTCTGAA R: GCTTCCGCTTCTCATCCTT	85	60
*HK2*	Hexokinase 2	NM_000189.4	F: AACAGCCTGGACGAGAGCA R: AGCAACCACATCCAGGTCAAA	135	58
*PKM2*	Pyruvate kinase M1/2	NM_002654.5	F: CGCATGCAGCACCTGATTG R: GCGGCGGAGTTCCTCAAATA	75	58
*PFK*	Phosphofructokinase	NM_001166686.2	F: CCTCCCCCAGCTTCCCG R: CATGGCTGCCTCCTAGCG	150	58
*LDHA*	Lactate dehydrogenase A	NM_005566.3	F: GATTCAGCCCGATTCCGTTA R: CATACAGGCACACTGGAATCT	107	56
*DVL2*	Dishevelled segment polarity protein 2	NM_004422.2	F: CTCCATCCTTCCACCCTAATG R: GACACTACTGACTCGGTTTCTG	76	58
*CTNNB1*	Catenin beta 1	NM_001904.3	F: CAGGTGGTGGTTAATAAGGCT R: CATCTGAGGAGAACGCATGATAG	90	60
*NFKB1*	Nuclear factor kappa B subunit 1	NM_003998.3	F: GGAAGTACAGGTCCAGGGTATAG R: CCATGCTTCATCCCAGCATTAG	107	58
*RPLPO*	Ribosomal protein lateral stalk subunit P0	NM_001002.3	F: ACGGATTACACCTTCCCACTTGCTGAAAAGGT R: AGCCACAAAGGCAGATGGATCAGCCAAG	69	65
*RPS17*	Ribosomal protein S17	NM_001021.6	F: AAGCGCGTGTGCGAGGAGATCG R: TCGCTTCATCAGATGCGTGACATAACCTG	87	65
*H3F3A*	H3 histone family member 3A	NM_002107.4	F: AGGACTTTAAAACAGATCTGCGCTTCCAGAG R: ACCAGATAGGCCTCACTTGCCTCCTGC	76	65

## Results

### Clinical and biochemical characteristics of the groups studied during pregnancy and postparum

Table 2 shown clinical characteristics of the different experimental groups both during and after pregnancy. At the first visit, the patients diagnosed as having GDM were slightly but significantly older than the NGT subjects and displayed significantly higher fasting and post-load glucose levels, insulin and HOMA-IR and lower QUICKI-IS compared to control subjects (*p* < 0.05). Additionally, maternal HbA1c level was significantly lower in the GDM group than NGT controls, although these values were within the normal range in both groups (<6%) (*p* < 0.05). No difference was noted between the NGT and GDM groups in the remaining clinical parameters.

**Table 2:** Clinical characteristics of the study participants at GDM diagnosis (the first visit) and postpartum at 3 months (the second visit) and at one year (the third visit).

	**1st visit**			**2nd visit**			**3rd visit**		
**Variable**	**NGT (*n* = 37)**	**GDM (*n* = 98)**	***p***	**group A1 (*n* = 8)**	**group B (*n* = 8)**	***p***	**group A2 (*n* = 12)**	**group C (*n* = 12)**	***p***
Age (year)	29.0 (26.0–31.8)	31.0 (28.0–34.3)	0.02*	33.5 (29.0–36.5)		NA	31.0 (27.5–35.8)		NA
Pre-pregnancy BMI (kg/m^2^)	23.0 (21.2–27.4)	25.5 (21.7–29.8)	0.163	22.2 (20.3–30.9)		NA	22.3 (20.6–29.6)		NA
Pregnancy BMI [kg/m^2^]	26.9 (25.2–31.3)	28.5 (24.5–32.8)	0.412	25.8 (21.8–31.8)		NA	28.0 (23.6–33.2)		NA
Body weight gain (kg)	8.5 (7.0–12.0)	7.7 (4.5–10.5)	0.051	11.0 (4.3–13.0)		NA	9.3 (4.8–12.4)		NA
SBP ([mmHg)	127.0 (114.0–134.0)	123.5 (114.5–132.3)	0.690	129.0 (121.5–131.0)	130.0 (116.0 –135.0)	0.999	129.0 (123.0–132.5)	ND	NA
DBP ([mmHg)	73.0 (70.0–78.0)	76.0 (69.8–83.0)	0.230	79.0 (69.0–85.0)	77.0 (76.0–90.0)	0.474	78.0 (70.5–83.5)	ND	NA
TGs (mg/dL)	207.5 (161.7–239.3)	208.2 (170.5–252.0)	0.467	216.2 (188.5–269.9)	95.5 (75.0–155.8)	0.016*	202.1 (134.3–260.8)	96.2 (51.3–130.6)	0.156
TC (mg/dL)	265.3 (229.6–293.5)	255.4 (231.2–290.3)	0.600	252.6 (229.5–272.5)	198.5 (175.9–226.4)	0.016*	239.1 (183.6–261.1)	194.0 (159.3–203.8)	0.156
HDL-C (mg/dL)	80.1 (64.3–95.0)	78.3 (66.9–89.5)	0.641	64.2 (58.9–71.9)	53.2 (40.1–75.3)	0.375	63.8 (56.8–74.5)	53.0 (42.5–75.3)	0.094
LDL-C (mg/dL)	143.0 (126.0–174.0)	139.0 (114.0–175.0)	0.569	138.5 (118.3 –168.5)	116.0 (106.8 –134.3)	0.297	124.0 (103.0–154.3)	102.0 (90.0–133.8)	0.563
HbA1C [%]	5.3 (5.1–5.5)	5.0 (4.8–5.3)	0.0002***	4.9 (4.6–5.9)	5.3 (5.0–5.7)	0.156	5.1 (4.8–5.3)	5.1 (5.0–5.3)	0.787
FPG (mg/dL)	80.0 (74.5 - 85.5)	86.0 (80.8–91.0)	0.0009***	81.0 (77.0–90.3)	83.0 (77.0–83.0)	0.441	87.5 (78.0–92.9)	92.0 (86.0–102.5)	0.916
OGTT 1-h glucose (mg/dL)	163.5 (144.8–174.0)	175.0 (151.0–188.0)	0.005**	186.0 (165.0–191.5)	ND	NA	167.0 (151.0–184.0)	ND	NA
OGTT 2-h glucose (mg/dL)	132.0 (110.5–144.0)	162.0 (151.8–170.3)	<0.0001***	153.0 (145.0 –174.0)	104.0 (83.8–133.5)	0.050*	157.0 (146.3–172.3)	107.0 (92.8–111.8)	0.031*
Insulin [µIU/mL]	8.0 (4.5–11.7)	10.7 (7.7–16.2)	0.007**	9.9 (6.8–13.5)	6.4 (4.3–12.9)	0.219	12.9 (6.7–15.3)	10.3 (4.7–18.8)	0.563
HOMA-IR	1.5 (0.8–1.8)	2.2 (1.6–3.6)	0.0003***	2.1 (1.2–2.9)	1.3 (0.9–2.9)	0.547	2.6 (1.3–3.6)	2.6 (1.0–4.9)	0.563
HOMA- *β* [%]	144.0 (87.0–245.0)	171.5 (121.0–249.3)	0.078	180.9 (128.2–290.1)	106.8 (94.4–182.1)	0.313	180.9 (150.6–261.8)	99.3 (77.4–183.0)	0.438
QUICKI-IS	0.36 (0.34–0.39)	0.3 (0.3–0.4)	0.003**	0.3 (0.3–0.4)	0.4 (0.3–0.4)	0.383	0.3 (0.3–0.4)	0.3 (0.3–0.4)	0.563
CRP (mg/dL)	2.8 (1.8–9.3)	3.9 (2.2–6.4)	0.251	3.3 (2.1–5.1)	1.1 (0.7–2.2)	0.016*	3.9 (2.0–5.1)	1.7 (0.5–2.3)	0.156

Notes.

Data are presented as median values with 25–75% interquartile range.

* *p* < 0.05.

** *p* < 0.01.

*** *p* < 0.001.

NGT vs. GDM as assessed by the Mann-Whitney U test and group A1 and A2 at the first visit vs. group B at the postpartum second visit or group C at the postpartum third visit as assessed by Paired Samples Wilcoxon Test.

NA not applicable ND not determined

When clinical parameters of the postpartum women at the second (the group B, *n* = 8) and third (the group C; *n* = 12) visits were compared to the same women with diagnosed GDM at the first study visit (the corresponding groups A1 and A2), there were significantly lower 2 h post-load glucose concentrations in both postpartum groups versus the corresponding diabetic groups (Table 2). In addition, the postpartum group B displayed significantly lower plasma TC, TGs, and CRP concentrations than the corresponding group A1 (Table 2).

**Figure 2:**
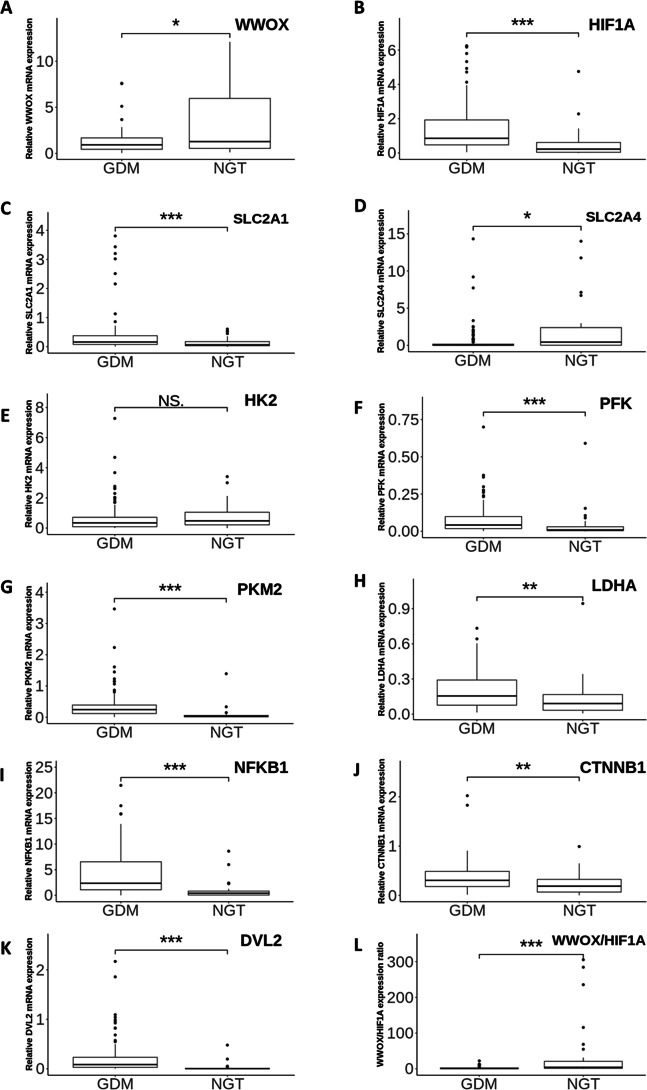
The gene expression profiles of *WWOX, HIF1A* and *WWOX/HIF1A*-related genes. Boxplots showing the relative leukocyte mRNA expression (the ratio of the target gene relative to the reference genes RPS17, RPLP0, H3F3A) of *WWOX* (A), *HIF1A* (B) and all WWOX/HIF-related genes including those involved in glucose transport (*SLC2A1* (C), *SLC2A4* (D)), glycolytic pathway (*HK2* (E), *PKM2* (F), *PFK* (G), *LDHA* (H)), Wnt pathway (*DVL2* (I), *CTNNB1* (J)), and inflammatory response (*NFKB1* (K)), along with the *WWOX/HIF1A* ratio (L) in the patients with GDM (*n* = 98) vs the subjects with NGT (*n* = 37). Data are expressed as median (indicated by horizontal bars) ± interquartile range (25–75%), ^∗^*p* < 0.05, ^∗∗^*p* < 0.01, ^∗∗∗^*p* < 0.001 as assessed by the Mann–Whitney U-test. NS, not significant.

### Gene expression studies during and after pregnancy

At the first visit, the relative mRNA expression of the 11 individual genes involved in the WWOX/HIF1A axis was investigated in leukocytes obtained from the patients with and without GDM. Quantitative RT-PCR expression data showed that leukocyte *HIF1A*, *PFK*, *PKM2*, *DVL2*, *NFKB1* ( all *p* < 0.0001 ), *SLC2A1* (*p* = 0.0004), *LDHA* (*p* = 0.0015)*,* and *CTNNB1* (*p* = 0.002) mRNA levels were significantly higher, whereas leukocyte *SLC2A4* expression was significantly lower (*p* = 0.0134) in the patients with GDM in comparison with the subjects with NGT (Fig. 2). There was also a tendency for lower leukocyte *WWOX* expression in the women with GDM *vs* the controls with NGT (*p* = 0.0406). As *HIF1A* and *WWOX* displayed opposing patterns of expression and their functional acting is known to be closely interrelated, we employed the *WWOX*/*HIF1A* expression ratio as an indicator of leukocyte transcriptional response of the two related genes to GDM. As shown in Fig. 2, the *WWOX*/*HIF1A* index was decreased by approximately 6.5-fold (*p* <0.0001) in the GDM group compared to the NGT group; thus it appears to be a better indicator of leukocyte transcriptional response of the two related genes to GDM than each of these two genes separately, as evidenced by an 1.4- fold decrease for *WWOX* and a 3.8-fold increase for *HIF1A* in the diabetic patients. Of note, the leukocyte *HK2* transcript level remained unchanged between the two groups (*p* > 0.05).

**Figure 3:**
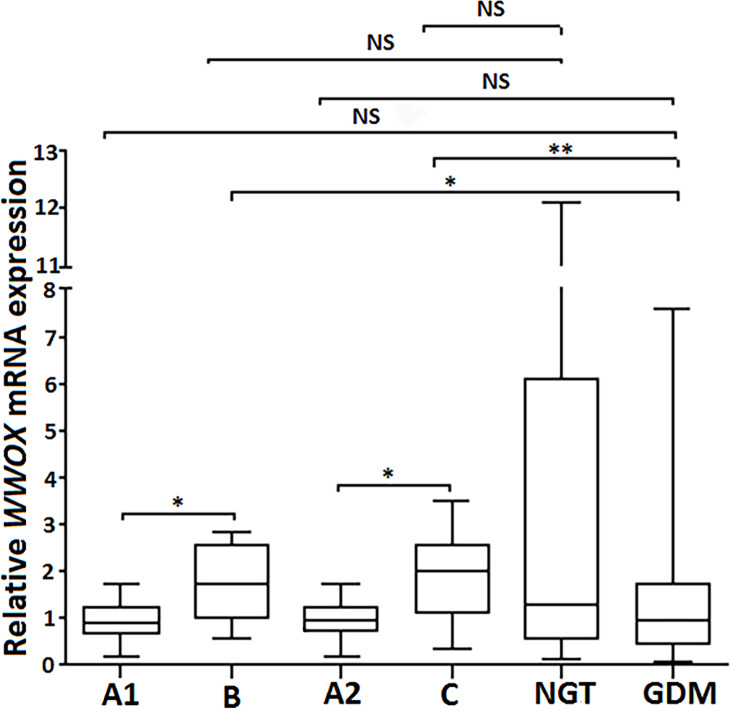
Postpartum leukocyte WWOX mRNA expression in the study groups. The relative leukocyte *WWOX* mRNA expression in the group B (at the second visit) and group C (at the third visit) vs corresponding diabetic groups A1 and A2 (pregnant women diagnosed as having GDM at the first study visit; *n* = 8 and *n* = 12, respectively) vs pregnant subjects who were classified as NGT (*n* = 37) and GDM (*n* = 98) at the first visit. Data are expressed as median (indicated by horizontal bars) ± interquartile range (25–75%), ^∗^*p* < 0.05, ^∗∗^*p* < 0.01, as assessed by the Wilcoxon Matched-Pairs Signed Rank Test for paired results and the Mann–Whitney *U* test for unpaired.

Subsequently, the leukocyte expression pattern of all the aforementioned genes was compared between patients from the postpartum groups B (at the second visit; *n* = 8) and C (at the third visit, *n* = 12) versus the corresponding control groups A1 and A2 (the same patients diagnosed as having GDM at the first study visit). As shown in Fig. 3. the patients from the postpartum groups B and C exhibited a 2-fold increase in leukocyte *WWOX* gene expression compared to the same women from the corresponding groups A1 and A2, respectively (*p* < 0.05), with no significant difference in *WWOX* expression between groups A1, A2 and GDM (all patients complicated by GDM included in the study; *n* = 98; *p* >0.05). Of note, leukocyte *WWOX* expression in the postpartum groups B and C was comparable with that found in the NGT group (control) at the first visit.

### Gene expression correlations

**Figure 4:**
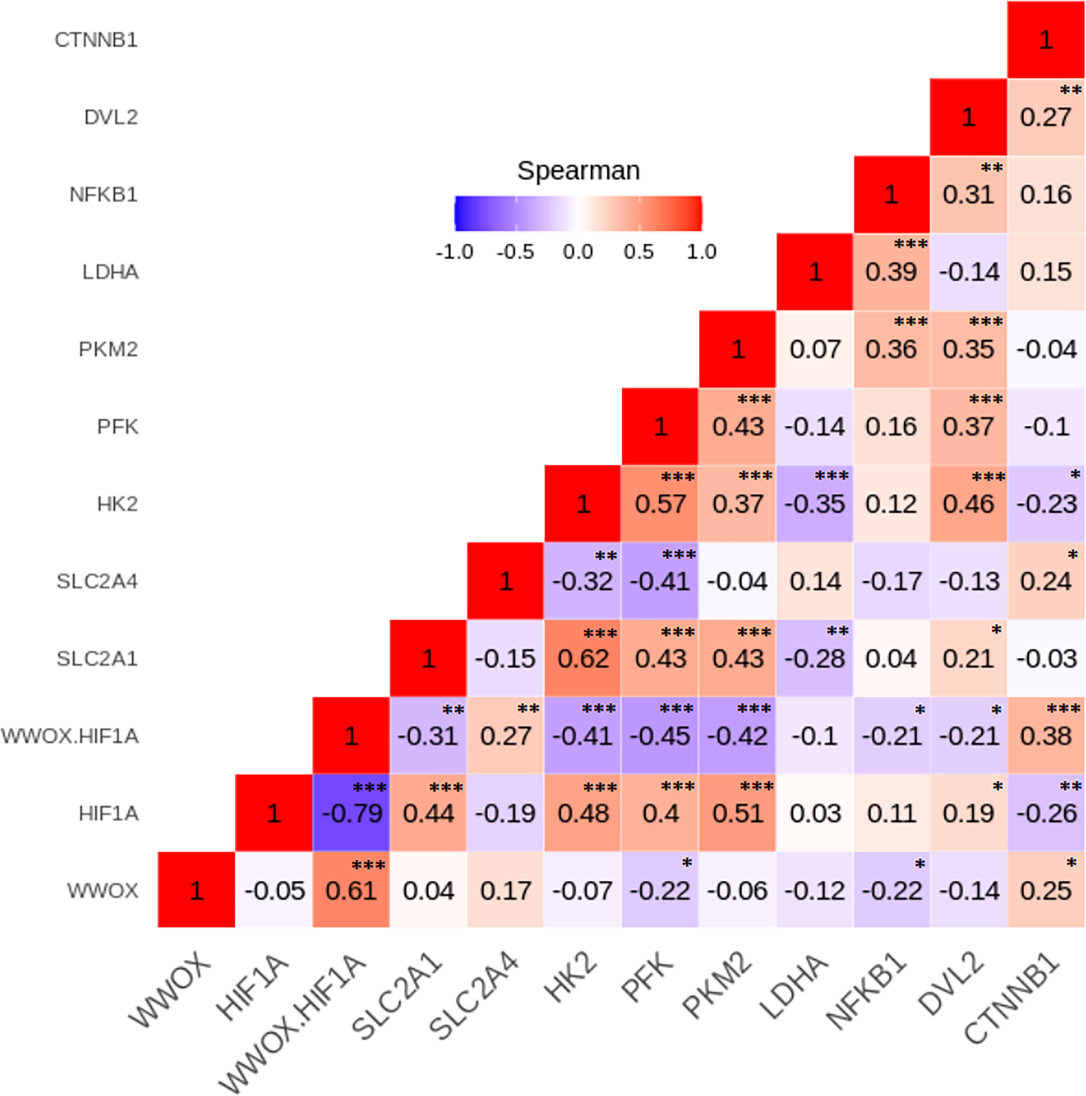
The Spearman’s correlation analysis of genes expression in the GDM group. The Spearman’s multiply correlation plot with R representing correlation coefficient and *p* the statistical significance (^∗^*p* < 0.05, ^∗∗^*p* < 0.01, and ^∗∗∗^*p* < 0.001) for the transcripts of interest in the GDM group. R was presented in different colors; the legend is the color range of different R values.

**Figure 5:**
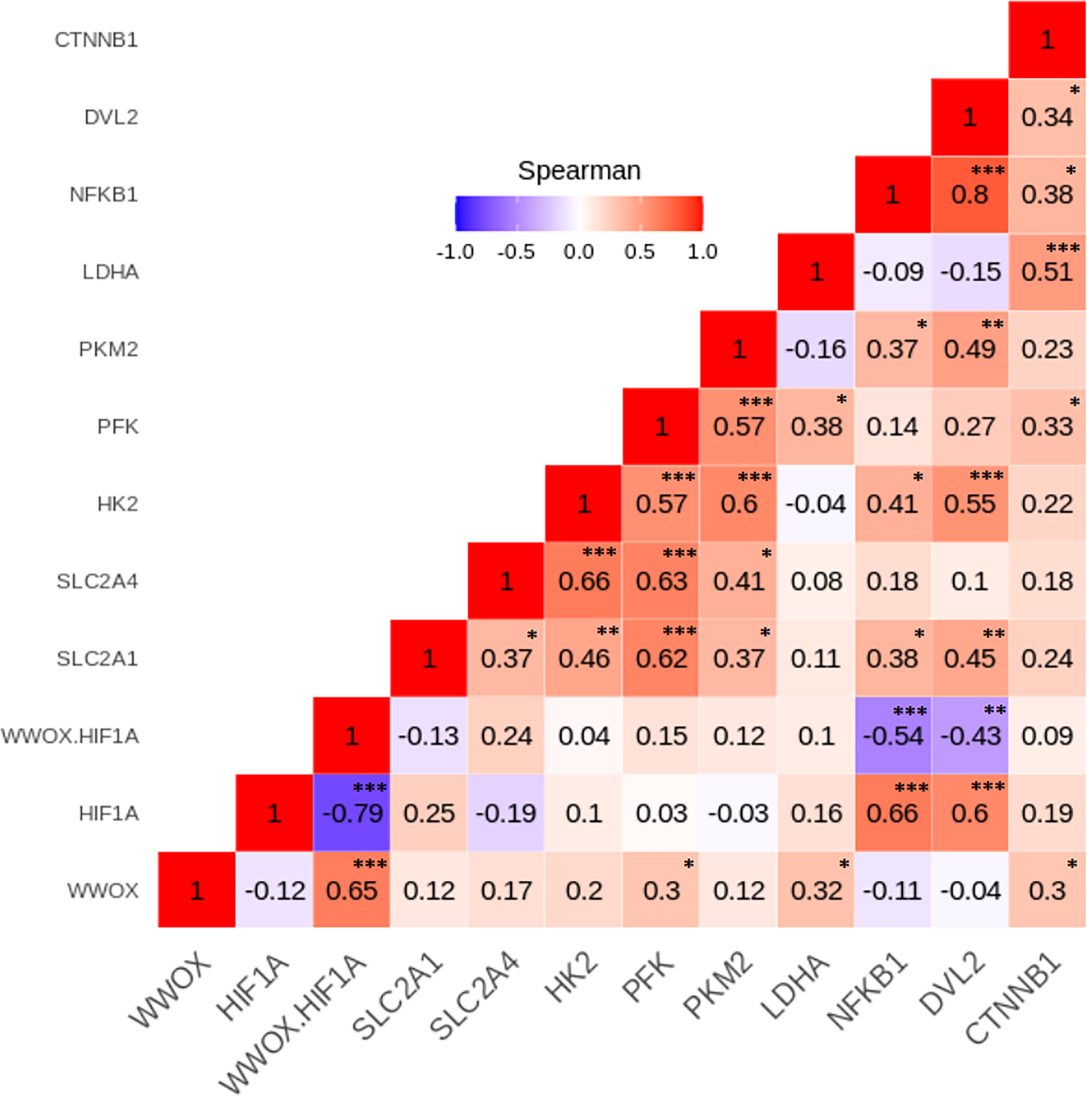
The Spearman’s correlation analysis of genes expression in the NGT group. The Spearman’s multiply correlation plot with R representing correlation coefficient and p the statistical significance (^∗^*p* < 0.05, ^∗∗^*p* < 0.01, and ^∗∗∗^*p* < 0.001) for the transcripts of interest in the GDM group. R was presented in different colors; the legend is the color range of different R values.

To investigate relationships between the leukocyte expression profiles of the 11 WWOX/HIF1-response genes among the GDM and NGT participants, the Spearman’s correlation analyses were made, and numerous significant positive and negative correlations between genes (*p* < 0.05) were identified in the two group studied (Figs. 4 and 5). In general, these analyses revealed that transcript levels for each of the genes in the *WWOX*/*HIF1* pathway share correlation with at least three other genes in the pathway in diabetic pregnancy, suggesting highly connected and co-regulated pathway. In the patients with GDM, many correlations were found between *WWOX/HIF1A* ratio and other genes: positively with *SLC2A4* (*R* = 0.27; *p* < 0.01) and *CTNNB1* ( *R* = 0.38; *p* < 0.001) and negatively with *NFKB1* ( *R* =  − 0.21; *p* < 0.05*)* and *DVL2* ( *R* =  − 0.21; *p* = 0.05), *SLC2A1* (*R* =  − 0.31; *p* = 0.01), *HK2* (*R* =  − 0.41)*, PFK* ( *R* =  − 0.45) and *PKM2* (*R* =  − 0.42; all *p* < 0.001*).* Additionally, there were weak but significant positive associations of *WWOX* with *CTNNB1* ( *R* = 0.25) and *SLC2A4* (*R* = 0.17) and negative with *PFK* (*R* =  − 0.23) and *NFKB1* (*R* =  − 0.22; all *p* < 0.05) and (*ii*) *HIF1A* associated negatively with *CTNNB1* (*R* =  − 0.26; *p* < 0.05) and *SLC2A4* (*R* =  − 0.19; *p* < 0.05) and positively with *SLC2A1* (*R* = 0.45; *p* <0.0001), *DVL2* (*R* = 0.19; *p* < 0.05) as well as several glycolytic genes, including *HK2* (*R* = 0.48), *PFK* (*R* = 0.40), and *PKM2* (*R* = 0.51; all *p* <0.0001).

In patients with NGT, the *WWOX/HIF1A* ratio is inversely correlated with *NFKB1* (*r* =  − 0.54; *p* < 0.001) and *DVL2* (*R* =  − 0.43; *p* < 0.01), whereas *HIF1A* transcript strongly associated with *NFKB1* and *DVL2* (*R* = 0.66 and *R* = 0.60, respectively; *p* < 0.001 for both). Furthermore, weak positive associations of *PFK*, *LDHA*, and *CTNNB1* were found for *WWOX* transcript (*R* = 0.30, *R* = 0.32, and *R* = 0.30, respectively; all *p* < 0.05).

The Spearman correlation coefficients for the remaining genes analyzed in the GDM and NGT groups are shown in the Figs. 4 and 5, respectively.

### Correlations between maternal metabolic parameters and the expression of WWOX/HIF-related genes

To establish whether clinical characteristics of the patients given in Table 2 is associated with the expression of *WWOX*/*HIF*-related genes, correlation analyses were made in the GDM and NGT groups at the time of GDM diagnosis and the postpartum period (3 months and 1 year).

**Figure 6:**
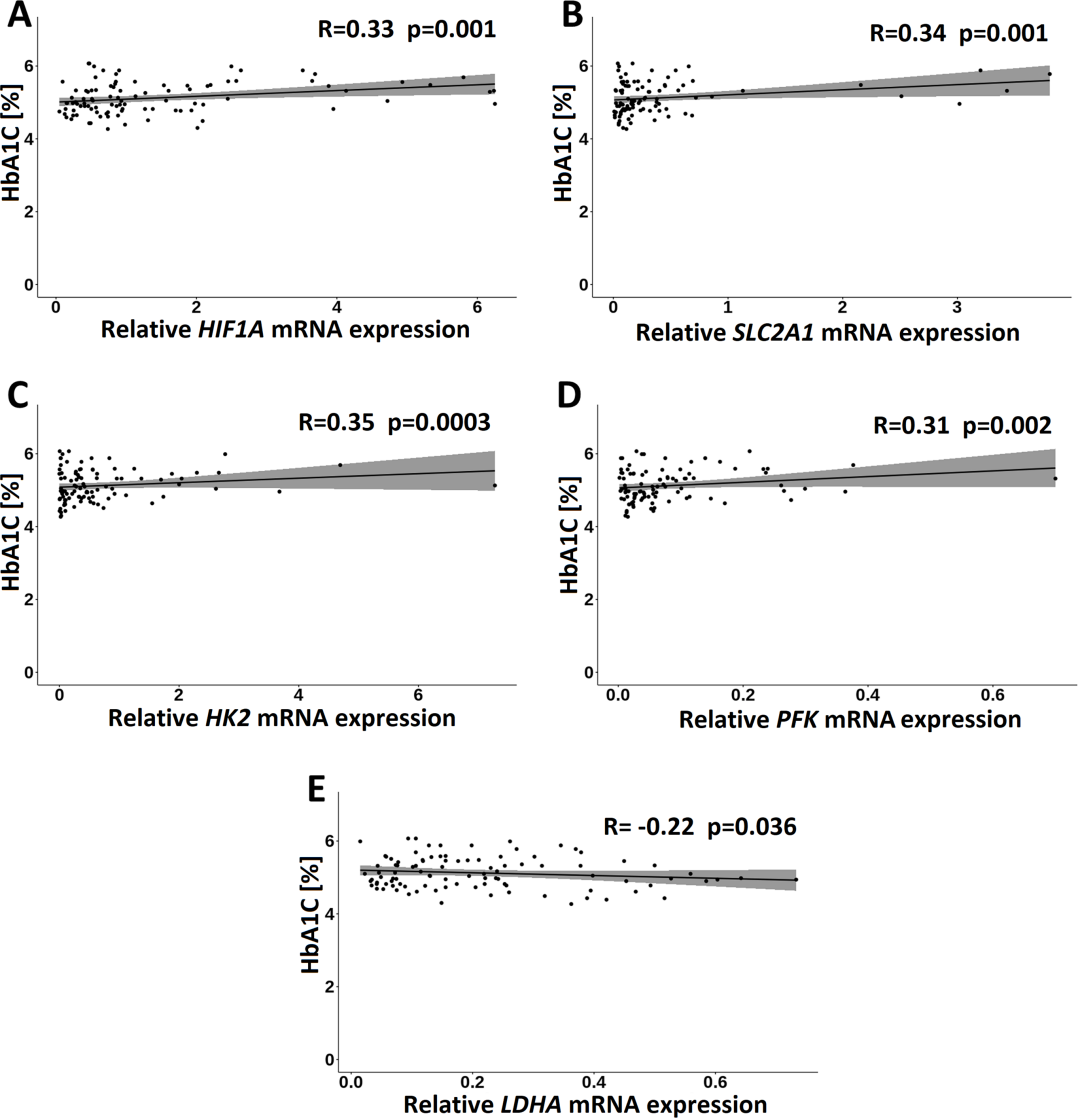
Correlations of HbA1C with *HIF1A* (A), *SLC2A1* (B), *HK2* (C), *PFK* (D) and *LDHA* (E) in the GDM group. Spearman correlation coefficients at *p* < 0.05 are shown.

In the GDM group, of the clinical measurements, only HbA1C correlated significantly and positively with *HIF1A* (*R* = 0.33; *p* = 0.001), *HK2* (*R* = 0.35; *p* = 0.004), *PFK* (*R* = 0.31; *p* = 0.002), and *SLC2A1* (*R* = 0.34; *p* = 0.001) transcripts and negatively with *LDHA* transcript (*R* =  − 0.27; *p* = 0.010) (Fig. 6).

In the NGT group, insulin and HOMA-IR index positively associated with *WWOX* expression (*R* = 0.52 and *R* = 0.53, respectively; *p* = 0.001 for both) and negatively with the *WWOX*/*HIF1A* ratio (*R* =  − 0.47; *p* = 0.004 for insulin and *R* =  − 0.44; *p* = 0.008 for HOMA-IR index). By contrast, QUICKI-IS index negatively correlated with *WWOX* expression (*R* =  − 0.52; *p* = 0.001) and positively with the *WWOX*/*HIF* ratio (*R* = 0.45; *p* = 0.007). There was also a weak inverse correlation between insulin and *HK2* (*R* =  − 0.34; *p* = 0.045) and *PFK* (*R* =  − 0.36; *p* = 0.034) expression (Table S1).

In the postpartum groups, no correlation was evident between metabolic phenotype of subjects and the expression of the genes studied (*p* > 0.05; data not shown).

## Discussion

**Figure 7:**
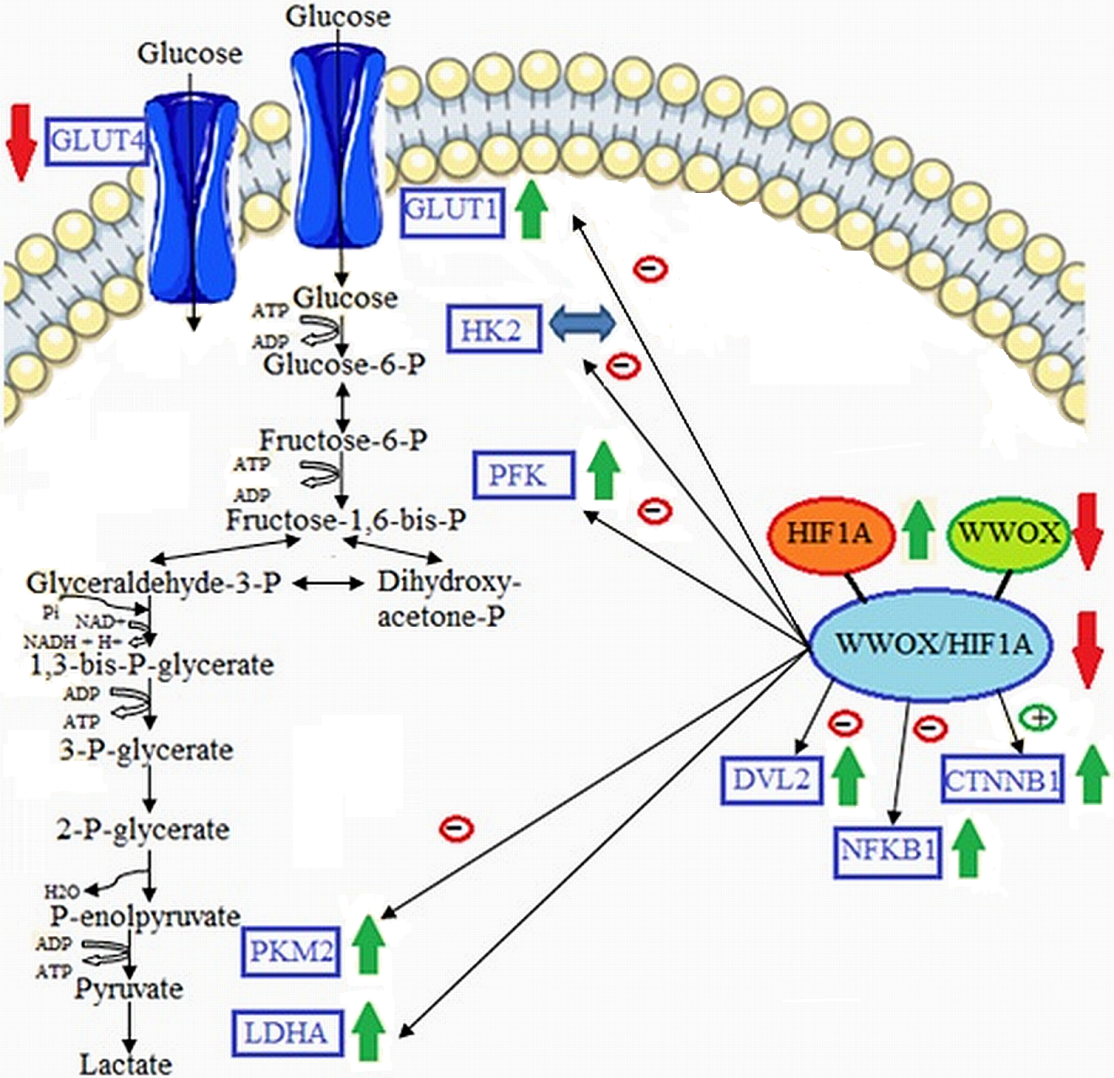
Proposed model of molecular connections between the components of the WWOX/HIF axis and the upregulation of glycolytic energy metabolism in leukocytes of diabetic patients. Based on free images (“https://smart.servier.com/smart_image/cell-membrane-14/”; “https://smart.servier.com/smart_image/channel-119/”) from Servier Medical Art (2020a) and Servier Medical Art (2020b); CC BY 3.0.

This study primary examined the GDM associated gene expression profiles of major components of the glucose metabolism as a consequence of differentiated *HIF1A/WWOX* pathway in peripheral blood leukocytes obtained from a clinically well-characterized Caucasian women with GDM at the time of GDM diagnosis and the postpartum period (3 and 12 months). Our results reported (i) the linkage of *WWOX*/*HIF1A*-driven transcriptional regulation of the glycolytic phenotype in leukocytes of GDM patients, (ii) a significant correlation between glycemic control and leukocyte gene expression of *HIF1A* and *WWOX* modulated expression of its target genes *HK2, PFK*, and *SLC2A1* that are involved in glycolysis, (iii) an important contribution of the *WWOX* gene to the pathogenesis of GDM by modulation HIF1α activity (Fig. 7).

Consistent with other studies (Czech et al., 2014), our GDM patients were characterized by hyperglycemia and insulin resistance, as reflected by greater HOMA-IR and lower QUICKI-IS indices compared to the control subjects. Several factors have been recognized to affect insulin resistance/sensitivity, including overweight or obesity, anti-hyperglycemic medications, diet, and physical activity (Genova et al., 2014); however, since parameters of obesity such as pre-pregnancy BMI (women diagnosed with GDM were slightly heavier than NGT women, but this difference was not statistically significant) and body weight gain were comparable between women with and without GDM in the present study, and furthermore, since our newly diagnosed GDM patients had not been in receipt of any the above-mentioned pharmacological and non-pharmacological treatment at the time of inclusion into the study, we may exclude their possible effect on the insulin resistance/sensitivity parameters in GDM women.

Although the molecular aspects of diet are not part of this work, it is worth mentioning that as was recently reported there is association between dietary patterns and gene expression features shown in, peripheral blood mononuclear cells (Christensen et al., 2020). What is more, HIF1α protein was shown increased in visceral adipose tissue (VAT) from the high fat diet mouse model and in VAT obtained from obese patients compared to those of normal-weight controls (Arcidiacono et al., 2020), and then hypoxia in obese adipose tissue may play a critical role in the impairment of peripheral insulin action and development of systemic insulin resistance. Unexpectedly, maternal HbA1c levels, reflecting glycemic status over the preceding 2–3 months, were significantly lower in the GDM than NGT group, although their values were within the normal range in both groups with an A1C<6.5%, based on the WHO and American Diabetes Association (ADA) guidelines for the diagnosis of diabetes in non-pregnant individuals (Gillett, 2009); *Use of Glycated Haemoglobin (HbA1c) in the Diagnosis of Diabetes Mellitus*, 2011; Standards of medical care in diabetes, 2018). Currently, no diagnostic threshold of HbA1c is not defined for GDM, and the reference values for HbA1c in different ethnic populations during pregnancy are still debated. This is because HbA1c may be influenced by different factors, especially variation in the life span of erythrocyte and iron deficiency. This latter disturbance in iron homeostasis, for reasons not yet understood, may increase the blood HbA1c level, independent of glycemia, even when anemia is absent (Brooks et al., 1980; Kim et al., 2010). This might explain higher maternal HbA1c levels in non-diabetic subjects *versus* GDM patients observed in our study. Unfortunately, no measurements of iron status were done in the pregnancies enrolled in this study. On the other hand, we cannot exclude the possibility that high glucose concentration in our GDM patients might shorten the life span of erythrocyte, which falsely lower HbA1c levels, as hyperglycemia itself has been shown to reduce erythrocyte survival through the mechanism that is still unknown (Virtue et al., 2004).

There is compelling evidence that hypoxia is strongly linked to human physiology and pathophysiology, with a particular role of HIF1α in these processes (Semenza, 2001). It is now widely accepted that HIF1α and its responsive genes encoding glycolytic enzymes and glucose transporters, among others, are upregulated in cancer cells as the consequence of metabolic shift of glucose metabolism from the more energetic efficient mitochondrial oxidative phosphorylation (OXPHOS) pathway to the less energetic efficient glycolytic pathway, even when oxygen level is adequate (the Warburg effect) (Xie & Simon, 2017). In the case of diabetes, which like cancer is characterized by a dysregulation of glucose metabolism, there are mixed results showing impaired (Catrina et al., 2004) or enhanced (Xiao et al., 2006; Isoe et al., 2010) HIF-1 pathway under high glucose conditions.

In the present study, we found a significantly higher leukocyte mRNA expression of both *HIF1A* and its target genes, including glucose transporter *SLC2A1* and the rate limiting glycolytic enzymes such as *PFK, PKM2*, and *LDHA* in the patients with GDM compared to the subjects with NGT, suggesting a transcriptional up-regulation of the glycolytic phenotype in leukocytes of the diabetic patients. This finding is in line with a previous study showing that hyperglycemia can induce the HIF1α-signaling pathway in the mesangial cells under in vitro conditions, with the increased expression of the HIF target genes *SLC2A1* and *HK2* in these cells (Isoe et al., 2010). The HK2 converts glucose into glucose 6-phosphate during the first rate limiting step of glycolysis, and its elevated expression has been implicated in promoting tumor growth and invasion (Anderson et al., 2017) as well as developing diabetic glomerulopathy (Isoe et al., 2010). In the present study, no significant change was found in leukocyte *HK2* gene expression profile between the GDM and NGT groups. In contrast to mentioned literature reports, Machado et al. stated a 50% reduction in *HK2* mRNA and HK2 protein in skeletal muscle of diabetic rats (Esteves et al., 2018). Authors suggest miRNAs participation in the HK2 repression in skeletal muscle of diabetic rats. What is more, Myatt (Muralimanoharan, Maloyan & Myatt, 2016) et al. research of *SLC2A1, HK2, PFK* and *LDHA* expression in placenta after cesarean section showed statistically significant upregulation only in GDM women treated with glyburide or insulin in comparison to NGT, no in GDM treated with diet alone. Nonetheless, our gene-gene Spearman correlations analysis revealed that both *HK2* and *PFK*, *PKM2*, and *SLC2A1* positively associated with *HIF1A*, but only in the GDM group, not the NGT group. Such correlation was stronger with *WWOX/HIF1A* suggesting that WWOX modulates HIF1A function similarly to cancer cells. This unique gene expression correlation pattern further supports the conception that glycolytic energy metabolism is upregulated in the diabetic patients. In the current study, the expression of *HIF1A*, *HK2*, and *PFK* transcripts was also found to display a positive correlation with maternal HbA1C in the GDM group, suggesting the enhanced *HIF1A* response in patients with a poor glycemic control. These results raised the question of how the late phase of maternal hyperglycemia might upregulate *HIF1A*, *HK2*, and *PFK*. Although the molecular mechanism(s) that underlies this association remain understood, we cannot rule out the possibility that hyperglycemia might regulate *HIF1A* expression through altering cellular redox status since high glucose has been proposed to increase the cytosolic ratio of free NADH/NAD+ in diabetic patients (the phenomenon defined as pseudohypoxia) (Williamson et al., 1993; Caniggia et al., 2000; Song, Yang & Yan, 2019). An increase in basal HIF-1α mRNA expression has also been observed in hearts of diabetic rats as well as in isolated non-diabetic rat hearts perfused with high glucose under non-hypoxic condition, confirming a pseudohypoxic state caused by hyperglycemia (Marfella et al., 2002). In this context, contrary to what was expected, *LDHA* transcript inversely correlated with maternal HbA1C level as well as *SLC2A1* and *HK2* transcripts. The protein encoded by *LDHA* [lactate dehydrogenase A] is a pivotal enzyme converting pyruvate into lactate in the final step in aerobic glycolysis, and its catalytic activity directly correlates with lactate production (Arriarán et al., 2015). Thus, our results stand in contrast to a previous study demonstrating the increased blood lactate concentration and lactate dehydrogenase activity in GDM patients that positively correlated with the HbA1c level (Nagalakshmi et al., 2016; Nadimi Barforoushi, Qujeq & Roudi, 2017). It should be kept in mind that the blood lactate level measured in the previous study reflects the whole body response to hyperglycemia, while our study concentrated on the transcriptional changes only in leukocytes.

Evidence has accumulated in recent years indicating that GDM is strongly linked to the impaired insulin signaling pathway and glucose uptake alterations mediated by GLUT4 transporter, which is known to act as an insulin-responsive glucose transporter. In the present study, a significant decrease in leukocyte *SLC2A4* mRNA expression was detected in hyperglycemic and insulin resistant women with GDM in compared with NGT controls. Similarly, previous studies demonstrated GLUT4 downregulation in skeletal muscle, adipose tissue, and placenta of pregnant women complicated by GDM, as the consequence of the dysfunction of insulin signaling (Colomiere et al., 2009; Colomiere, Permezel & Lappas, 2010). Thus, this may partially account for the insulin resistance occurring in our GDM patients. On the other hand, we cannot rule out the possibility that *SLC2A1* overexpression which is accompanied by *SLC2A4* downregulation in the patients with GDM might be a compensatory process to maintain glucose influx despite of elevated blood glucose in these subjects.

The tumor suppressor WWOX has been implicated in glucose homeostasis through regulating HIF1α and its target genes (Abu-Remaileh & Aqeilan, 2014). More recently, the importance of functional crosstalk between WWOX and HIF1α has also been reported in mice with Wwox-specific ablation in skeletal muscle, that is an animal model associated to the phenotype resembling metabolic syndrome (Abu-Remaileh et al., 2019). We also showed a linkage of WWOX to HIF1α at their transcriptional levels in leukocytes of the patients with GDM. In contrast to the significant upregulation of *HIF1A* leukocyte *WWOX* expression was downregulated in diabetic pregnancy *versus* normal controls, and it inversely associated with leukocyte *PFK1* transcript in these patients. The *PFK* gene encodes phosphofructokinase 1 (PFK1), a key rate-limiting enzyme of glycolysis, which catalyzes the irreversible conversion of fructose-6-phosphate (F-6-P) and ATP into fructose-1,6-bisphosphate (F-1,6-BP) and ADP. Thus, these findings, along with negative correlations observed in the GDM group between the *WWOX*/*HIF1A* ratio and several glycolytic genes driven by HIF-1α (*SLC2A1*, *HK2*, *PFK*, and *PKM2*) support an important contribution of *WWOX* downregulation toward enhanced glycolysis. It should be noted that although neither *WWOX* expression nor the *WWOX/HIF1A* ratio correlated with metabolic phenotypes of diabetic patients, they associated with insulin resistance/sensitivity parameters of pregnancies with NGT, suggesting a plausible role of WWOX in progressive insulin resistance, which is the hallmark of normal glucose regulation during pregnancy. In fact, pregnancy is normally accompanied by progressive insulin resistance that begins near mid-pregnancy and progresses through the third trimester, predominantly as the result of increasing production of placental hormones. In this state, most pregnant women maintain normal glucose tolerance through a compensatory increase in insulin secretion by pancreatic β cells.

The limitation of our research is the fact that it was not possible to investigate the reason for the decrease in WWOX expression in GDM patients. It is known that increasing evidence implicate altered DNA methylation in the pathophysiology of gestational diabetes mellitus (GDM) (El Hajj et al., 2013; Reichetzeder et al., 2016; Dias et al., 2019) and a possible general defect in DNA methylation in diabetes is suggested (Maier & Olek, 2002; Enquobahrie et al., 2015). WWOX gene is classified among the differentially methylated T2D susceptibility genes in adipose tissue (Nilsson et al., 2014) and one of the targets of DNA methylation affecting the Warburg effect (Zhu et al., 2020). Recent reports point to an important role of WWOX as one of the differentially expressed genes (DEGs) in pancreatic, muscle, and adipose tissue in type 2 diabetics, simultaneously overlapping with the subnetwork of genes responsible for insulin secretion and insulin activity, measured by HOMA- β and HOMA-IR, respectively. The authors suggest that these genes, including WWOX, which are significantly associated with the intermediate glycemic traits of HOMA-IR and HOMA- β are likely to be help to identify the subnetworks of T2D protein-protein interaction that can be targeted for understanding pathogenic mechanisms that lead to disease (Saxena et al., 2020).

Strikingly, we found significantly higher postpartum *WWOX* expression in women with prior GDM compared to that displayed by the same women with diagnosed GDM, but its level was comparable to that observed in normal pregnancy. Hence, leukocyte *WWOX* downregulation seen in our GDM patients may be a unique feature of diabetic pregnancy, implicating WWOX in the pathogenesis of GDM. However, there was a relatively small population size of postpartum patients with prior GDM (*n* = 8 and *n* = 12 at 3 months and 1 year postdelivery, respectively) compared to the number of GDM women who participated in the study (*n* = 98), limiting our statistical significance. Although all GDM women participating in this study were recommended for postpartum glucose testing, only a very small number of diabetic patients came to the OGTT in the postpartum period. It is in line with a common global trend in extremely low the follow-up rate of postpartum glucose testing in women with a history of GDM (Koning et al., 2016; Pastore et al., 2018). Many factors contribute to this phenomenon, including lack of time, difficulties with child care, less risk awareness to develop diabetes in the future, or transport difficulties, among others (Bernstein et al., 2016; Van Ryswyk et al., 2016). Thus, developing more satisfactory strategies are required for improving women’s participation in postpartum screening.

It is now widely accepted that exaggerated inflammation is closely associated with the development and course of GDM, and numerous inflammatory mediators participating in these processes have been recognized so far (Zhao et al., 2011; Gomes et al., 2013). Among them, the nuclear factor-κB (NFκB) is thought to be a crucial regulator of the immune and inflammatory responses since it is involved in inducible expression of a variety of cellular genes encoding pro-inflammatory cytokines, chemokines, and adhesion molecule (Kuzmicki et al., 2013). In agreement with previously published data (Feng et al., 2016), we found the increased *NFKB1* mRNA level in hyperglycemic and insulin resistant patients with GDM, suggesting that this change may be related to the pathophysiology of hyperglycemia and insulin resistance that are evident in women with GDM. Further, we observed that *NFKB1* expression correlated inversely with the expression *WWOX* and *SLC2A4* as well as the *WWOX/HIF1A* ratio and positively with the expression of HIF-1α-responsive genes such as *PKM2* and *LDHA* in the GDM women, clearly pointing to a crosstalk between *NFKB1* upregulation, *WWOX* downregulation, impaired GLUT4-mediated intracellular transport of glucose, and enhanced glycolytic pathway in the condition of diabetic pregnancy; however, whether these associations have functional relevance for the development of GDM remains to be determined. It is noteworthy that although our gene correlation analyses failed to demonstrate the existence of an relationship of *NFKB1* with *HIF1A* in the women with GDM, both genes were significantly upregulated in these patients, implying their relevance in the setting of human diabetic pregnancy. In support of this hypothesis, a previous study revealed a close relationship between the placental HIF-1α and NF-kB upregulation in a mouse model of GDM, which leads to an excessive inflammatory response (Li, Chen & Li, 2013). In this context, a strong positive correlation between the expression of *HIF1A* and *NFKB1* found in our healthy pregnant women is surprising, thereby the explanation for this association remains not resolved and warrants further studies.

The Wnt/β-catenin signaling has been implicated in numerous physiological and pathophysiological processes, and its importance in glucose homeostasis through regulating pancreatic beta cell proliferation and mass as well as insulin secretion has been described in transgenic mouse models (Rulifson et al., 2007). Several lines evidence also point to the occurrence of polymorphisms in the gene encoding TCF7L2, an important downstream target of the canonical Wnt signaling pathway, that associate with an increased risk of the development of T2DM (Grant et al., 2006; Florez, 2007). Numerous studies in cultured cells and animal models suggest that the exposure to high glucose may activate the Wnt/β-catenin signaling pathway, but the regulation of some components of this pathway is still poorly understood (Liu, Liang & Yang, 2015). In our study, the expression of the *CTNNB1* and *DVL2* genes, encoding β-catenin and dishevelled 2 (DVL-2) protein, respectively, was significantly increased in leukocytes of hyperglycemic and insulin resistant patients with GDM compared to that noted in normal controls, thereby suggesting that the activation of the Wnt signaling pathway may be linked with the metabolic phenotype of diabetic patients. Further, in patients with GDM, *DVL2* correlated positively with *HIF1A* and its target genes, including *HK2*, *PFK*, *PKM2*, and *SLC2A1*, and negatively with the *WWOX/HIF1A* index, whereas there were inverse correlations in the expression between *CTNNB1* and *HIF1A* and *HK2*. Known is interaction between WWOX and DVL2 in the cytoplasmic compartment and its role as inhibitor of the Wnt/B-catenin pathway by sequestering *DVL* proteins in the cytoplasmic compartment to inhibition B-catenin stabilization and transcription activation (Bouteille et al., 2009), which confirms the increased expression of *DVL2* and *CTNNB1* with reduced *WWOX* expression in the leukocytes of GDM patients with a simultaneous negative correlation of *WWOX/HIF1A* with *DVL2.* Therefore, increased *CTNNB1* expression and a positive correlation with *WWOX/HIF1A* may be a compensatory effect as a reaction to inhibitory influence of *WWOX*.

Any discrepancies in our results in relation to the literature data may also result from the fact that leukocytes are examined as a whole population of different cells. Probably the analysis of the expression differences in leukocytes subsets would explain the observed variability, as the literature data indicate statistically significant differences in the number of individual fractions of white blood cells between the GDM and NGT groups (Hope, Ifeanyi & Braxton, 2019; Sargin et al., 2016).

## Conclusions

In conclusion, this study demonstrated that WWOX downregulation was accompanied by a transcriptional up-regulation of the glycolytic phenotype in leukocytes of diabetic patients, which positively associated with glycemic control in these subjects. In addition, the WWOX gene is proposed as an important contributor to the pathogenesis of GDM. However, the validity of these findings need to be confirmed in larger studies with more statistical power and with the analysis of protein expression of the components of the WWOX/HIF1A axis in leukocytes of GDM subjects.

##  Supplemental Information

10.7717/peerj.10604/supp-1 Supplemental Information 1 The Spearman’.s correlation between maternal metabolic parameters and the expression of WWOX/HIF-related genes R represents the correlation coefficient and p the statistical significance (^∗^*p* < 0.05, ^∗∗^*p* < 0.01, and ^∗∗∗^*p* < 0.001).

10.7717/peerj.10604/supp-2 Supplemental Information 2 The primary raw data acquired by the qRT-PCR system - plate 1 Quantitative RT-PCR (RT-qPCR) raw data results for all the studied genes (*WWOX, HIF1A*, *NFKB1, SLC2A1, SLC2A4*, *HK2, PKM2, PFK, LDHA*, *DVL2,* and *CTNNB1*) and the Ribosomal Protein Lateral Stalk Subunit P0 (*RPLPO),* 40S ribosomal protein S17 (*RPS17*), and H3 Histone Family Member 3A (*H3F3A*) used as the housekeeping genes for internal normalization. Results of reactions performed in duplicate on a LightCycler 480 II (Roche Diagnostics GmbH, Germany). The Universal Human Reference RNA (Agilent, USA) was used as a calibrator.

10.7717/peerj.10604/supp-3 Supplemental Information 3 The primary raw data acquired by the qRT-PCR system - plate 2 Quantitative RT-PCR (RT-qPCR) raw data results for all the studied genes (*WWOX, HIF1A*, *NFKB1, SLC2A1, SLC2A4*, *HK2, PKM2, PFK, LDHA*, *DVL2,* and *CTNNB1*) and the Ribosomal Protein Lateral Stalk Subunit P0 (*RPLPO),* 40S ribosomal protein S17 (*RPS17*), and H3 Histone Family Member 3A (*H3F3A*) used as the housekeeping genes for internal normalization. Results of reactions performed in duplicate on a LightCycler 480 II (Roche Diagnostics GmbH, Germany). The Universal Human Reference RNA (Agilent, USA) was used as a calibrator.

10.7717/peerj.10604/supp-4 Supplemental Information 4 The primary raw data acquired by the qRT-PCR system - plate 3 Quantitative RT-PCR (RT-qPCR) raw data results for all the studied genes (*WWOX, HIF1A*, *NFKB1, SLC2A1, SLC2A4*, *HK2, PKM2, PFK, LDHA*, *DVL2,* and *CTNNB1*) and the Ribosomal Protein Lateral Stalk Subunit P0 (*RPLPO),* 40S ribosomal protein S17 (*RPS17*), and H3 Histone Family Member 3A (*H3F3A*) used as the housekeeping genes for internal normalization. Results of reactions performed in duplicate on a LightCycler 480 II (Roche Diagnostics GmbH, Germany). The Universal Human Reference RNA (Agilent, USA) was used as a calibrator.

10.7717/peerj.10604/supp-5 Supplemental Information 5 The primary raw data acquired by the qRT-PCR system - plate 4 Quantitative RT-PCR (RT-qPCR) raw data results for all the studied genes (*WWOX, HIF1A*, *NFKB1, SLC2A1, SLC2A4*, *HK2, PKM2, PFK, LDHA*, *DVL2,* and *CTNNB1*) and the Ribosomal Protein Lateral Stalk Subunit P0 (*RPLPO),* 40S ribosomal protein S17 (*RPS17*), and H3 Histone Family Member 3A (*H3F3A*) used as the housekeeping genes for internal normalization. Results of reactions performed in duplicate on a LightCycler 480 II (Roche Diagnostics GmbH, Germany). The Universal Human Reference RNA (Agilent, USA) was used as a calibrator.

10.7717/peerj.10604/supp-6 Supplemental Information 6 The primary raw data acquired by the qRT-PCR system - plate 5 Quantitative RT-PCR (RT-qPCR) raw data results for all the studied genes (*WWOX, HIF1A*, *NFKB1, SLC2A1, SLC2A4*, *HK2, PKM2, PFK, LDHA*, *DVL2,* and *CTNNB1*) and the Ribosomal Protein Lateral Stalk Subunit P0 (*RPLPO),* 40S ribosomal protein S17 (*RPS17*), and H3 Histone Family Member 3A (*H3F3A*) used as the housekeeping genes for internal normalization. Results of reactions performed in duplicate on a LightCycler 480 II (Roche Diagnostics GmbH, Germany). The Universal Human Reference RNA (Agilent, USA) was used as a calibrator.

10.7717/peerj.10604/supp-7 Supplemental Information 7 The primary raw data acquired by the qRT-PCR system - plate 6 Quantitative RT-PCR (RT-qPCR) raw data results for all the studied genes (*WWOX, HIF1A*, *NFKB1, SLC2A1, SLC2A4*, *HK2, PKM2, PFK, LDHA*, *DVL2,* and *CTNNB1*) and the Ribosomal Protein Lateral Stalk Subunit P0 (*RPLPO),* 40S ribosomal protein S17 (*RPS17*), and H3 Histone Family Member 3A (*H3F3A*) used as the housekeeping genes for internal normalization. Results of reactions performed in duplicate on a LightCycler 480 II (Roche Diagnostics GmbH, Germany). The Universal Human Reference RNA (Agilent, USA) was used as a calibrator.

10.7717/peerj.10604/supp-8 Supplemental Information 8 The primary raw data acquired by the qRT-PCR system - plate 7 Quantitative RT-PCR (RT-qPCR) raw data results for all the studied genes (*WWOX, HIF1A*, *NFKB1, SLC2A1, SLC2A4*, *HK2, PKM2, PFK, LDHA*, *DVL2,* and *CTNNB1*) and the Ribosomal Protein Lateral Stalk Subunit P0 (*RPLPO),* 40S ribosomal protein S17 (*RPS17*), and H3 Histone Family Member 3A (*H3F3A*) used as the housekeeping genes for internal normalization. Results of reactions performed in duplicate on a LightCycler 480 II (Roche Diagnostics GmbH, Germany). The Universal Human Reference RNA (Agilent, USA) was used as a calibrator.

10.7717/peerj.10604/supp-9 Supplemental Information 9 The primary raw data acquired by the qRT-PCR system - plate 8 Quantitative RT-PCR (RT-qPCR) raw data results for all the studied genes (*WWOX, HIF1A*, *NFKB1, SLC2A1, SLC2A4*, *HK2, PKM2, PFK, LDHA*, *DVL2,* and *CTNNB1*) and the Ribosomal Protein Lateral Stalk Subunit P0 (*RPLPO),* 40S ribosomal protein S17 (*RPS17*), and H3 Histone Family Member 3A (*H3F3A*) used as the housekeeping genes for internal normalization. Results of reactions performed in duplicate on a LightCycler 480 II (Roche Diagnostics GmbH, Germany). The Universal Human Reference RNA (Agilent, USA) was used as a calibrator.

10.7717/peerj.10604/supp-10 Supplemental Information 10 The primary raw data acquired by the qRT-PCR system - plate 9 Quantitative RT-PCR (RT-qPCR) raw data results for all the studied genes (*WWOX, HIF1A*, *NFKB1, SLC2A1, SLC2A4*, *HK2, PKM2, PFK, LDHA*, *DVL2,* and *CTNNB1*) and the Ribosomal Protein Lateral Stalk Subunit P0 (*RPLPO),* 40S ribosomal protein S17 (*RPS17*), and H3 Histone Family Member 3A (*H3F3A*) used as the housekeeping genes for internal normalization. Results of reactions performed in duplicate on a LightCycler 480 II (Roche Diagnostics GmbH, Germany). The Universal Human Reference RNA (Agilent, USA) was used as a calibrator.

10.7717/peerj.10604/supp-11 Supplemental Information 11 The primary raw data acquired by the qRT-PCR system - plate 10 Quantitative RT-PCR (RT-qPCR) raw data results for all the studied genes (*WWOX, HIF1A*, *NFKB1, SLC2A1, SLC2A4*, *HK2, PKM2, PFK, LDHA*, *DVL2,* and *CTNNB1*) and the Ribosomal Protein Lateral Stalk Subunit P0 (*RPLPO),* 40S ribosomal protein S17 (*RPS17*), and H3 Histone Family Member 3A (*H3F3A*) used as the housekeeping genes for internal normalization. Results of reactions performed in duplicate on a LightCycler 480 II (Roche Diagnostics GmbH, Germany). The Universal Human Reference RNA (Agilent, USA) was used as a calibrator.

10.7717/peerj.10604/supp-12 Supplemental Information 12 The primary raw data acquired by the qRT-PCR system - plate 11 Quantitative RT-PCR (RT-qPCR) raw data results for all the studied genes (*WWOX, HIF1A*, *NFKB1, SLC2A1, SLC2A4*, *HK2, PKM2, PFK, LDHA*, *DVL2,* and *CTNNB1*) and the Ribosomal Protein Lateral Stalk Subunit P0 (*RPLPO),* 40S ribosomal protein S17 (*RPS17*), and H3 Histone Family Member 3A (*H3F3A*) used as the housekeeping genes for internal normalization. Results of reactions performed in duplicate on a LightCycler 480 II (Roche Diagnostics GmbH, Germany). The Universal Human Reference RNA (Agilent, USA) was used as a calibrator.

## Additional Information and Declarations

### Data Availability

The following information was supplied regarding data availability: The raw data are available in the Supplemental Files.
