# Supplementary material for: Identification of a novel association for the WWOX/HIF1A axis with gestational diabetes mellitus (GDM)
Source: PeerJ. 2021 Jan 14;9:e10604. doi: 10.7717/peerj.10604 (PMC7811782; doi:10.7717/peerj.10604)
Supplement: Supplemental Information 1 — R represents the correlation coefficient and p the statistical significance (∗p < 0.05, ∗∗p < 0.01, and ∗∗∗p < 0.001). [file peerj-09-10604-s001.docx]

| **GDM** | | | | | | | | | |
| --- | --- | --- | --- | --- | --- | --- | --- | --- | --- |
| ***Gene/Parameter*** | **R and  p-values** | ***LDHA*** | ***PFK*** | ***HK2*** | ***SLC2A*** | ***SLC2A1*** | ***HIF1A*** | ***WWOX*** | ***WWOX/HIF1A*** |
| ***HbA1C [%]*** | R value | **-0.27** | **0.31** | **0.35** | 0.20 | **0.34** | **0.33** | NS | NS |
|  | *p*-value | **0.010**** | **0.002**** | **0.004*** | 0.053 | **0.001***** | **0.001***** |  |  |

| **NGT** | | | | | | | | | |
| --- | --- | --- | --- | --- | --- | --- | --- | --- | --- |
| ***Insulin [µIU/mL]*** | R value | NS | **-0.36** | **-0.34** | NS | NS | NS | **0.52** | **-0.47** |
|  | *p*-value |  | **0.034*** | **0.045*** |  |  |  | **0.001**** | **0.004**** |
| ***HOMA-IR*** | R value | NS | NS | NS | NS | NS | NS | **0.53** | **-0.44** |
|  | *p*-value |  |  |  |  |  |  | **0.001**** | **0.008**** |
| ***QUICKI-IS*** | R value | NS | NS | NS | NS | NS | NS | **-0.52** | **0.45** |
|  | *p*-value |  |  |  |  |  |  | **0.001**** | **0.007**** |

NS, not significant
